# Mixtures of Macro and Micronutrients Control Grape Powdery Mildew and Alter Berry Metabolites

**DOI:** 10.3390/plants11070978

**Published:** 2022-04-04

**Authors:** Lior Gur, Yigal Cohen, Omer Frenkel, Ron Schweitzer, Meir Shlisel, Moshe Reuveni

**Affiliations:** 1 Shamir Research Institute, University of Haifa, Haifa 3498838, Israel; liogur@gmail.com (L.G.); mreuveni@research.haifa.ac.il (M.R.); 2 Faculty of Life Sciences, Bar-Ilan University, Ramat Gan 5290000, Israel; 3 Department of Plant Pathology and Weed Research, Agricultural Research Organization, The Volcani Center, Rishon LeZion 7528809, Israel; omerf@volcani.agri.gov.il; 4 Analytical Chemistry Laboratory, Tel-Hai College, Qiryat Shemona 1220800, Israel; ronschwei@gmail.com (R.S.); shlisel@telhai.ac.il (M.S.); 5 STK Bio-Ag Technologies Ltd., Petach Tikva 4951447, Israel

**Keywords:** *Erysiphe necator*, bio-stimulants, fertilizers therapy, metabolomics, secondary metabolites, antioxidants, integrated pest management, *Vitis vinifera*

## Abstract

Powdery mildew caused by the fungus *Erysiphe necator* is a major grape disease worldwide. It attacks foliage and berries and reduces yield and wine quality. Fungicides are mainly used for combating the disease. Fungicide resistance and the global requisite to reduce pesticide deployment encourage the use of environment-friendly alternatives for disease management. Our field experiments showed that the foliar application of the potassium phosphate fertilizer Top-KP+ (1-50-33 NPK) reduced disease incidence on leaves and clusters by 15–65% and severity by 75–90%, compared to untreated vines. Top-KP+ mixed with Nanovatz (containing the micronutrients boron (B) and zinc (Zn)) or with TruPhos Platinum (a mixture containing N, P_2_O_5_, K_2_O, Zn, B, Mg, Fe, Mn, Cu, Mo, and CO) further reduced disease incidence by 30–90% and disease severity by 85–95%. These fertilizers were as effective as the fungicide tebuconazole. Tank mixtures of fertilizers and tebuconazole further enhanced control efficacy in the vineyards. The modes of action of fertilizers in disease control were elucidated via tests with grape seedlings, microscopy, and berry metabolomics. Fertilizers applied preventively to the foliage of grape seedlings inhibited powdery mildew development. Application onto existing mildew colonies plasmolyzed mycelia and conidia and arrested the development of the disease. Berries treated with fertilizers or with a fungicide showed a significant increase in anti-fungal and antioxidant metabolites. Twenty-two metabolites, including non-protein amino acids and carbohydrates, known for their anti-fungal and bioactive effects, were significantly upregulated in grapes treated with fertilizers as compared to grapes treated with a fungicide, suggesting possible indirect activity against the pathogen. Esters and organic acids that contribute to wine quality were also upregulated. We conclude that integrating macro and micronutrients in spray programs in commercial vineyards shall control powdery mildew, reduce fungicide deployment, delay the buildup of fungicide resistance, and may improve wine quality.

## 1. Introduction

Grapevine is one of the most important crops worldwide, in relation to the production of both wine and table grapes. Several pests and diseases may affect the grapevine. Thus, an intensive pesticide schedule is often required to meet production standards [1]. The grape powdery mildew disease, caused by the fungal pathogen *Erysiphe necator* (syn. *Uncinula necator*) (Schw.) Burr., is one of the most important and destructive disease in vineyards worldwide. In wine grapes, cluster infection before or shortly after bloom may result in poor fruit set, considerable crop loss, and decrease in wine quality [2]. Disease management relies mainly on using multiple sprays per season of protectant and systemic fungicides, with 6–8 applications during years with favorable epidemic conditions [1,3]. Among the widely employed fungicides against grapevine powdery mildew are the strobilurins, quinone outside inhibitors (QoIs), demethylation inhibitors (DMIs), succinate dehydrogenase inhibitor (SDHI), and sulfur [3,4,5]. However, the development of fungicide-resistant strains of powdery mildew pathogens has been reported for 40 years [6]. Strains of *E. necator* with reduced sensitivity to QoIs, DMIs, and even the relatively new fungicide metrafenone have been reported worldwide in vineyards [7,8,9]. Because resistant strains survive for long periods, the risk of reinforcing a resistant population by re-application of ineffective fungicides is very high [10]. Therefore, alternative measures are urgently needed to combat powdery mildew in the vineyards. Reduced pesticide levels in food crops, concern for a healthy environment, and, often, the unavailability of commercially acceptable resistant cultivars emphasizes the need for alternative disease control methods. One such method is the foliar application of fertilizers that serve as ‘biocompatible’ fungicides [11,12]. Such fertilizers may also serve as a partial alternative for soil fertilization, avoiding some of the negative effects to the environment of leaching nutrients to the groundwater [13]. Foliar fertilizers have some advantages compared with soil-applied fertilizers. These include uptake by the leaves and rapid translocation to other organs, as demonstrated in grape seedlings [14]. Fertilizers are applied to foliage at optimum timing and concentrations according to the crop’s needs at different growth stages, as demonstrated with potassium in grapes [15]. The mixing of macro and micronutrients exploits synergistic effects between different nutrients, as shown in several field crops [16].

Previous studies demonstrated that foliar application of potassium silicate reduced powdery mildew on grape leaves [17]. Reuveni and co-workers showed that the foliar application of phosphate salts controlled powdery mildew in cucumber, roses, mango, apple, nectarine, and grapes [13,18,19]. The efficacy of the foliar application of micronutrients, such as Mn, Zn, B, and Si, against plant pathogens, including powdery mildews, has also been demonstrated [20,21,22]. However, the efficacy of the combinations of macro and micronutrients was rarely examined.

Secondary metabolites are important players in plant defense response. Studies have reported on the relationship between grape metabolic profile and resistance to pathogens [23,24]. Changes in metabolic profiles were induced in grapes by its pathogens, *Plasmopara viticola* [25] or *E. necator* [26]. Several studies reported effects of soil fertilization on the berry metabolic profile and wine composition [27,28,29,30]. The effect of the foliar application of nitrogen on grape berry quality was also reported [31,32,33]. However, limited information is available on the effect of the foliar application of potassium, phosphorus, or micronutrient mixture on the grape berry metabolic profile, and no data are available on the relationships between these nutrients, metabolic effects, and grape powdery mildew infection.

Using foliar fertilizers against pathogens may also affect the quality of the grapes and thus be important to the wine industry. Only limited data are available on such effects. Foliar treatments with biostimulants, hormones, or nitrogen compounds in vineyards were reported to modify grape and wine composition [32,33,34,35].

The present study was undertaken to: (a) evaluate the efficacy of foliar sprays of mixtures of macro and micronutrients in controlling powdery mildew in field-grown grapevines in comparison with recommended fungicides; and (b) investigate the modes of action of such nutrients on conidial germination and disease development in grape seedlings and berry metabolomics in the field.

## 2. Results

### 2.1. Effect on Powdery Mildew Development in Vineyards

During field trial 1 conducted in 2018, the first symptoms of powdery mildew were observed only on clusters of untreated control vines about one week after the fourth application of nutrient mixtures and fungicides, at BBCH-75 (Berries pea-sized, bunches hang). In disease rating conducted six days after the sixth application at BBCH-77 (berries beginning to touch), disease was apparent in all treatments, in relatively high incidence. However, the percentage of infected cluster area (disease severity) was significantly higher (86.4%) among the untreated controls as compared to fungicide and nutrient mixtures treated vines (1.6–4.5%) (*p* < 0.0001) (Table 1). Powdery mildew was less intensive in the second trial conducted in 2019 (Eshtaol vineyard) in which disease incidence and severity on control untreated vines reached 35% and 10.3%, respectively, at the end of the trial (Table 2). Top KP+ combined with either micronutrient mixture was more effective in reducing disease incidence than Top KP+ alone, compared to untreated vines, and was as effective as the fungicides Abir (quinoxyfen) and Heliosulfur in controlling powdery mildew (Table 2). Results of trial 3 in 2019 in Meron vineyard showed that, when applied alone, Top KP+ was less effective than Top KP+ with the micronutrient mixtures, or the fungicides, in reducing disease incidence and severity. Disease incidence and severity on control untreated vines were high and reached 100% and 33.6%, respectively. While Top KP+ reduced disease incidence and severity to 88% and 7.4% respectively, the mixtures with TruPhos or Nanovatz reduced disease incidence and severity to 68.5% and 4.8%, or 60% and 3.8%, respectively (Table 2). Nevertheless, in all three trials, Top KP+ in combinations with TruPhos or Nanovatz provided similar levels of disease control as both leading synthetic fungicides (Table 1 and Table 2).

The last two trials (4 and 5) conducted in 2020 in the Judean foothills region aimed to examine the integrated management of powdery mildew, including Top KP+ plus TruPhos or Nanovatz either applied alone, or each in combination with Folicur (tebuconazole), or alternated with Folicur. Results of trial 4 (Eshtaol vineyard) showed that despite the high disease pressure (Incidence of 73.5% and 98.3%, and severity of 26.2% and 55.8% on leaves and clusters, respectively), treatments of Top KP+ mixed with micronutrients were as effective as the fungicide Folicur, and similar results were obtained when mixtures were applied in alternation with Folicur. Compared to untreated vines, all treatments reduced disease incidence on leaves and clusters by 85–98% and disease severity by 97–100% (Table 3). However, because of the high efficacy level, no improvement of the tank-mixture of Top KP+ plus TruPhos or Nanovatz with Folicur, either on leaves or clusters, was observed compared to each nutrient mixture alone (Table 3). A reduced rate of Top KP+ to 0.5% in the mixture with TruPhos yielded reduced efficacy (Table 3). The second trial conducted in 2020 (trial 5 in Mazkeret-Batya vineyard) showed that the combination of Top KP+ plus TruPhos or Nanovatz with Folicur improved disease control on leaves and clusters, compared to each nutrient mixture alone (Table 4). Disease incidence was 98.8% and 69.4%, and severity was 55.8% and 36.3% on leaves and clusters of untreated vines, respectively. Tank mixture of Top KP+ plus TruPhos with Folicur reduced disease incidence and severity on leaves by 64% and 85%, respectively, and on clusters by 62% and 89%, respectively, compared to Top KP+ plus TruPhos alone. The combination of Top KP+ plus Nanovatz with Folicur reduced disease incidence and severity on leaves by 78% and 97%, respectively, and on clusters by 78% and 90%, respectively, compared to Top KP+ plus Nanovatz alone (Table 4).

### 2.2. Effects of Nutrients and Fungicide on Must Oenological Parameters and Berry Weight

In both cultivars, ‘Carignan’ (red) and ‘Riesling’ (white) collected from two vineyards, macro and micronutrients applications did not affect must oenological parameters. The pH and Brix values were comparable to those obtained in grapes treated with fungicides or untreated grapes (Table S1). The average weight of berries collected from vines treated with nutrient mixtures or the fungicide Folicur was not significantly higher than that of berries collected from control untreated vines (Table S1).

### 2.3. Effect of Nutrients on Berry Metabolites

#### 2.3.1. *Vitis vinifera* cv. Riesling

**Figure 1:**
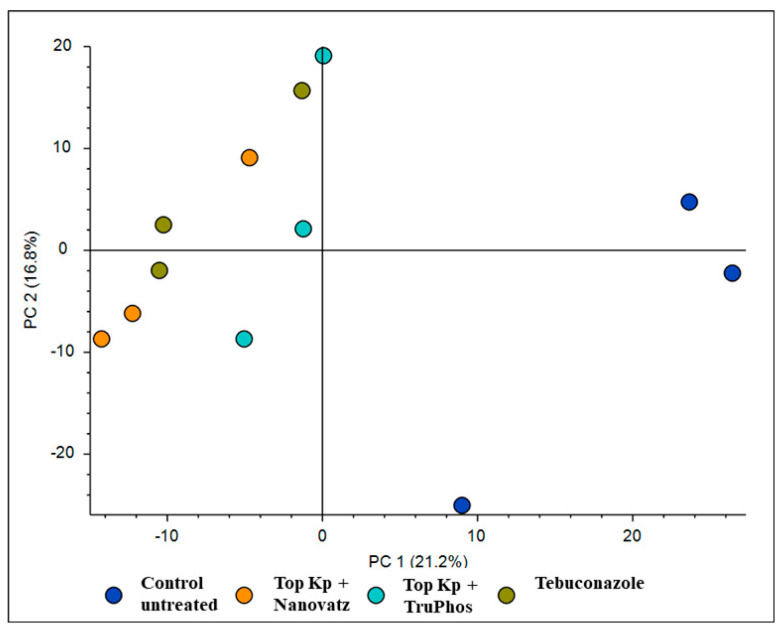
PCA analysis for 840 compounds identified by LC-MS/MS in cv. Riesling grape skin tissues treated by the fungicide Folicur (tebuconazole) 0.02%, Top KP+ 1% plus Nanovatz 0.1%, or Top KP+ 1% plus TruPhos 0.25% and untreated grapes.

**Figure 2:**
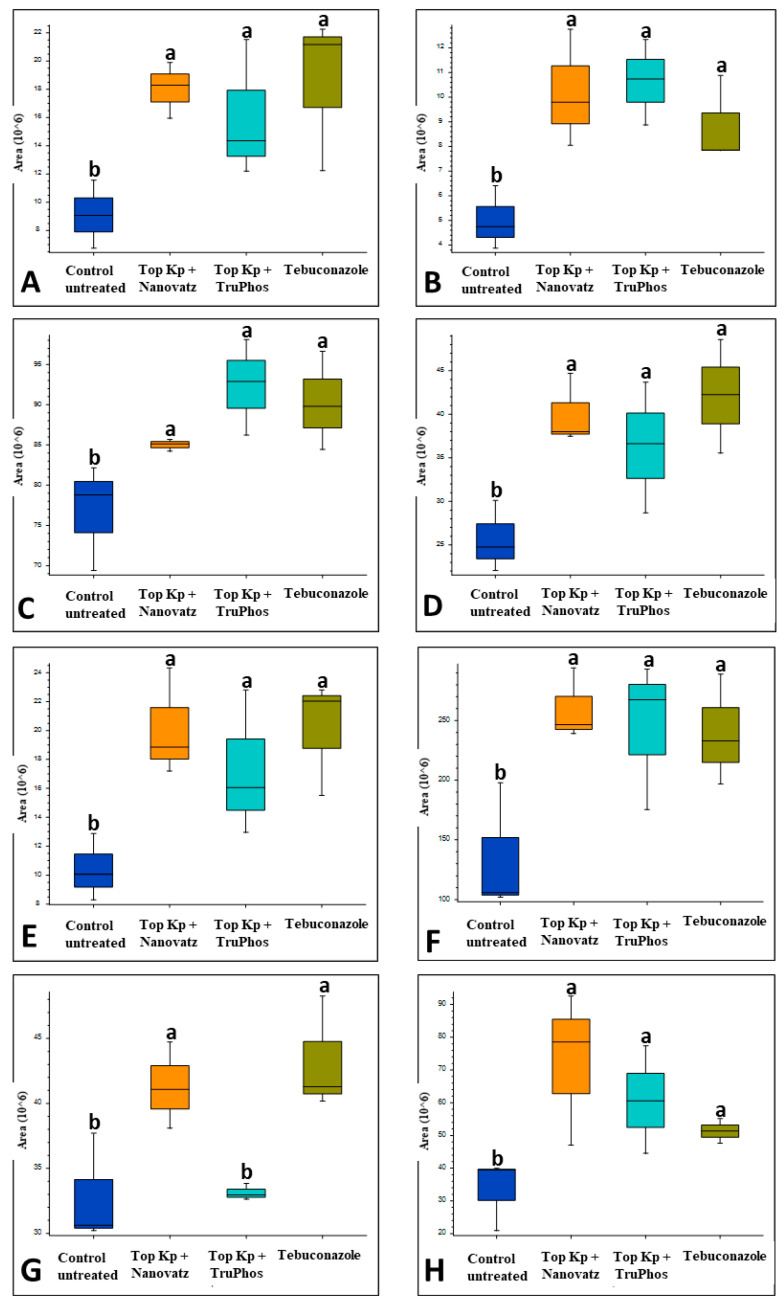
Relative amounts (peak area) of metabolites that were up-produced in grape skins of cv. Riesling vines treated with five foliar spays (starting at 14 April until 3 June) of the fungicide Folicur (tebuconazole) 0.02%, or the fertilizers Top KP+ 1% plus Nanovatz 0.1%, Top KP+ 1% plus TruPhos 0.25%, compared to untreated control vines. Analysis was conducted using LC–MS/MS. (**A**) the peptide Leu-Arg, (**B**) the peptide dihydroxy-phenylalanine, (**C**) the peptide Boc-Ser-OH, (**D**) the peptide Boc-Gln-OH, (**E**) the peptide L-gamma-Glutamyl-L-leucine, (**F**) Pheophorbide A, (**G**) Tuliposide B, (**H**) Nocardicin E. Different letters represent significant differences (*p* <  0.05, Tukey-Kramer HSD test).

**Figure 3:**
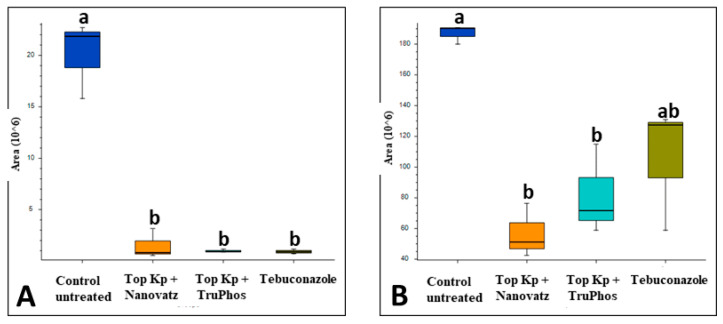
Relative amounts (peak area) of metabolites that were up-produced in grape skins of cv. Riesling untreated control vines, compared to vines treated with five foliar spays (starting at 14 April until 3 June) of the fungicide Folicur (tebuconazole) 0.02%, or the fertilizers Top Kp+ 1% plus Nanovatz 0.1%, Top KP+ 1% plus TruPhos 0.25%. Analysis was conducted using LC–MS/MS. (**A**) Cis-Resveratrol, (**B**) Catechin 3-O-gallate. Different letters represent significant differences (*p* <  0.05, Tukey-Kramer HSD test).

PCA analysis of 840 compounds identified by LC-MS/MS in cv. Riesling grape skin tissues revealed that the compound composition in untreated grapes was significantly different (*p* < 0.05) from those in grapes treated by the fungicide Folicur, Top KP+ plus Nanovatz, or Top KP+ plus TruPhos (Figure 1). Most of the metabolites were recognized by formulas, and only several compounds were annotated according to existing databases (Table S2). Among metabolites that were significantly upregulated in the sprayed grapes, some were annotated as dipeptides (Table 5). The content of dipeptides, such as Leu-Arg, dihydroxy-phenylalanine, Boc-Ser-OH, Boc-Gln-OH, and L-gamma-Glutamyl-L-leucine, was significantly higher in grapes treated with macro and micronutrients, or fungicide, than in untreated grapes (Figure 2A–E). The amount of chlorophyll breakdown product Pheophorbide A, known as an antioxidant, and Tuliposide B and Nocardicin E, known to have potent antimicrobial activity (Table 5), were also significantly higher in grapes treated with macro and micronutrients, or fungicide, than in untreated grapes (Figure 2F–H). The two metabolites cis-Resveratrol (stilbenoid) and Catechin 3-O-gallate (flavan-3-ol), which are related to plant stress, were significantly upregulated (*p* < 0.0005 and *p* < 0.05, respectively) in the severely mildew infected berries of the control untreated grapes (Figure 3).

**Figure 4:**
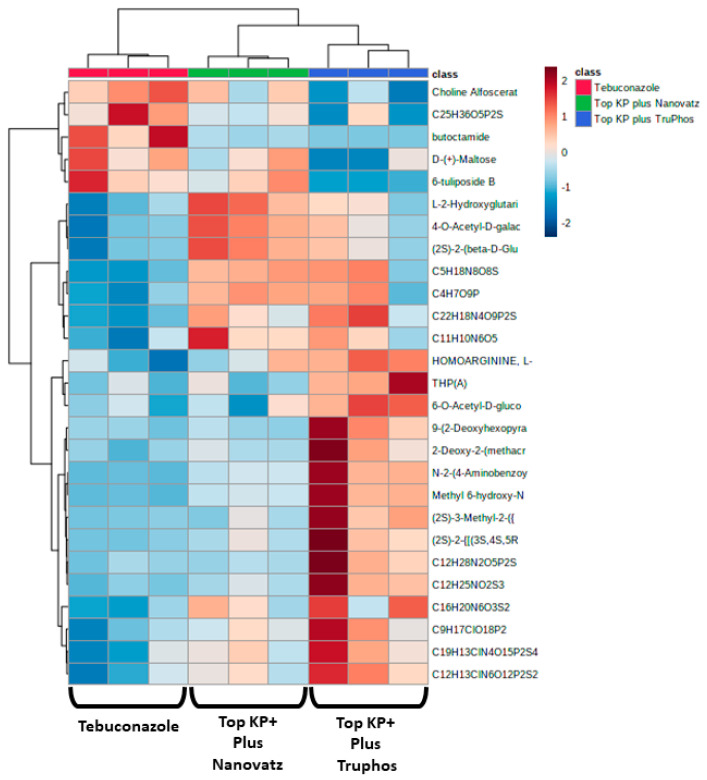
Heatmap and Hierarchical Cluster Analysis graph for all metabolites significantly (*p* < 0.05, Tukey-Kramer HSD test) up-produced or down-produced in grape skins of cv. Riesling in the fungicide Folicur (tebuconazole) 0.02% treatment samples and the fertilizers Top KP+ 1% plus Nanovatz 0.1%, or Top KP+ 1% plus TruPhos 0.25% treatments samples. The *x*-axis represents three repetitions of each treatment.

**Figure 5:**
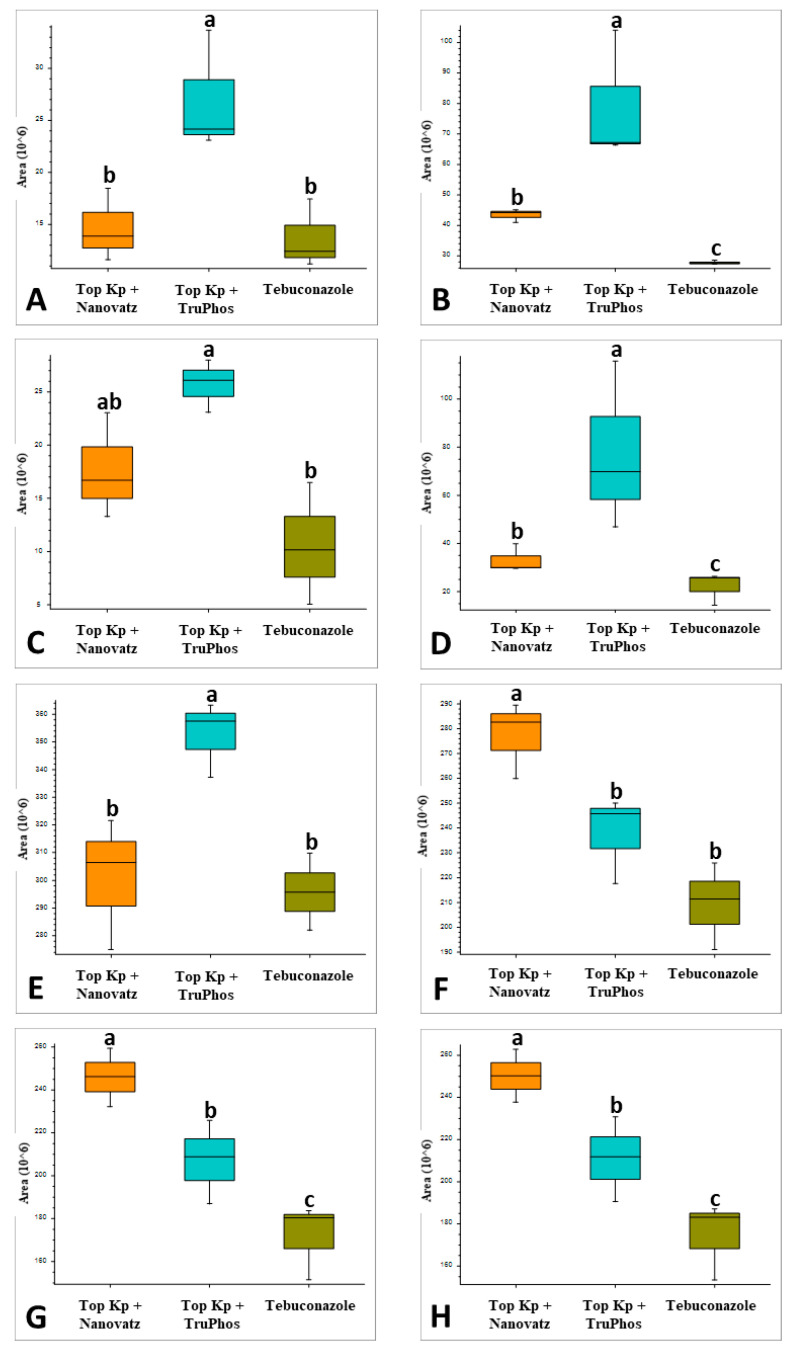
Relative amounts (peak area) of metabolites detected in grape skins of cv. Riesling vines treated with five foliar spays (starting at April 14 until June 3) of the fungicide Folicur (tebuconazole) 0.02%, Top KP+ 1% plus Nanovatz 0.1%, or Top KP+ 1% plus TruPhos 0.25%. Analysis was conducted using LC–MS/MS. (**A**) THP(**A**), (**B**) N~2~-(4-Aminobenzoyl) arginine, (**C**) L-Homoarginine, (**D**) 2-Deoxy-2-(methacryloylamino)-D-glucopyranose, (**E**) 6-O-Acetyl-D-glucose, (**F**) L-2-Hydroxyglutaric acid, (**G**) (2S)-2-(beta-D-Glucopyranosyloxy) succinic acid, (**H**) 4-O-Acetyl-D-galacturonic acid. Different letters represent significant differences (*p* <  0.05, Tukey-Kramer HSD test).

Since untreated control grapes were severely infected with powdery mildew (56% severity), we compared metabolites composition between grapes treated with the fungicide Folicur to those treated with the macro and micronutrients, which were all almost healthy (<0.3%, severity) (Table 3). Results showed that 22 metabolites were significantly (*p* < 0.05) upregulated in grapes treated with the fertilizers Top KP+ plus Nanovatz or Top KP+ plus TruPhos, as compared to grapes treated with the fungicide Folicur (Figure 4). Among 22 metabolites that were significantly upregulated, eight were annotated (Table 5). The amount of THP(A), N~2~-(4-Aminobenzoyl) arginine, non-protein amino acid L-Homoarginine, and carbohydrates 2-Deoxy-2-(methacryloylamino)-D-glucopyranose and 6-O-Acetyl-D-glucose were significantly higher (*p* < 0.05) in grapes treated with Top KP+ plus TruPhos compared to grapes treated with Folicur. However, lower, or no significant differences were observed between grapes treated with Top KP+ plus Nanovatz, compared to grapes treated with Folicur (Figure 5A–E). The amount of L-2-Hydroxyglutaric acid was significantly higher (*p* < 0.04) in grapes treated with Top KP+ plus Nanovatz as compared to grapes treated with Folicur (Figure 5F). The amount of the organic acids (2S)-2-(beta-D-Glucopyranosyloxy) succinic acid and 4-O-Acetyl-D-galacturonic acid was significantly high (*p* < 0.04) in grapes treated with Top KP+ plus TruPhos and even higher in grapes treated with Top KP+ plus Nanovatz compared to grapes treated with Folicur (Figure 5G,H).

#### 2.3.2. *Vitis vinifera* cv. Carignan

**Figure 6:**
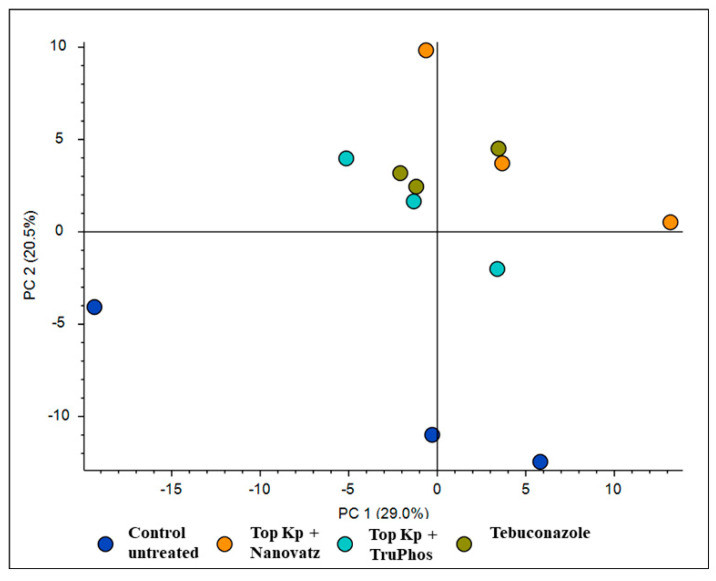
PCA analysis for 205 compounds identified by LC-MS/MS in cv. Carignan grape skin tissues treated with six foliar spays (starting at 6 April until 8 June) of the fungicide Folicur (tebuconazole) 0.02%, Top KP+ 1% plus Nanovatz 0.1%, or Top KP+ 1% plus TruPhos 0.25% and untreated grapes.

**Figure 7:**
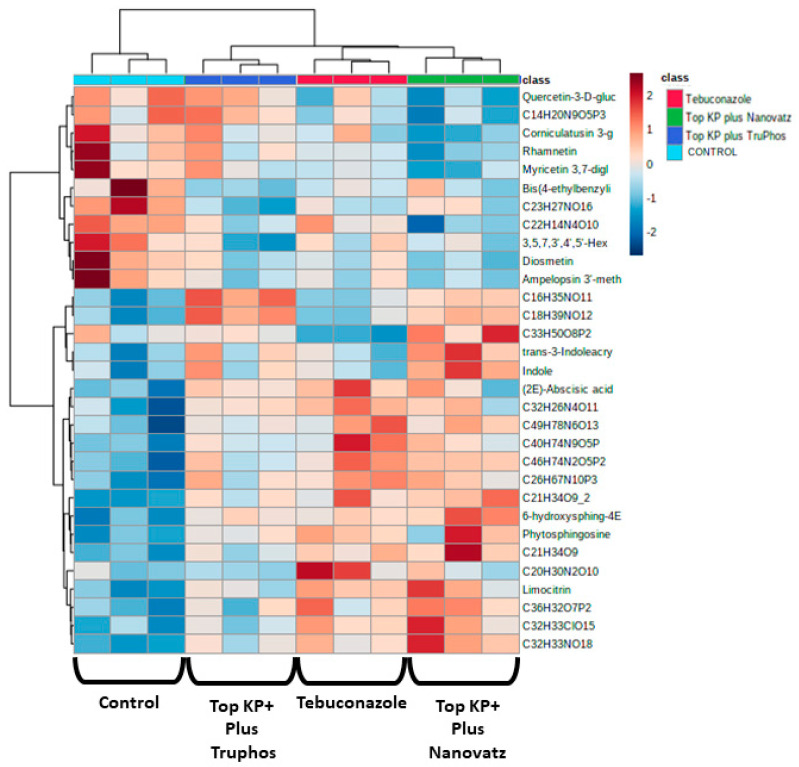
Heatmap and Hierarchical Cluster Analysis graph for all metabolites significantly (*p* < 0.05, TukeyKramer HSD test) up-produced or down-produced in grape skins of cv. Carignan in the fungicide Folicur (tebuconazole) 0.02%, the fertilizers Top KP+ 1% plus Nanovatz 0.1%, or Top KP+ 1% plus TruPhos 0.25% and the control untreated treatments samples. The *X*-axis represents three repetitions of each treatment.

**Figure 8:**
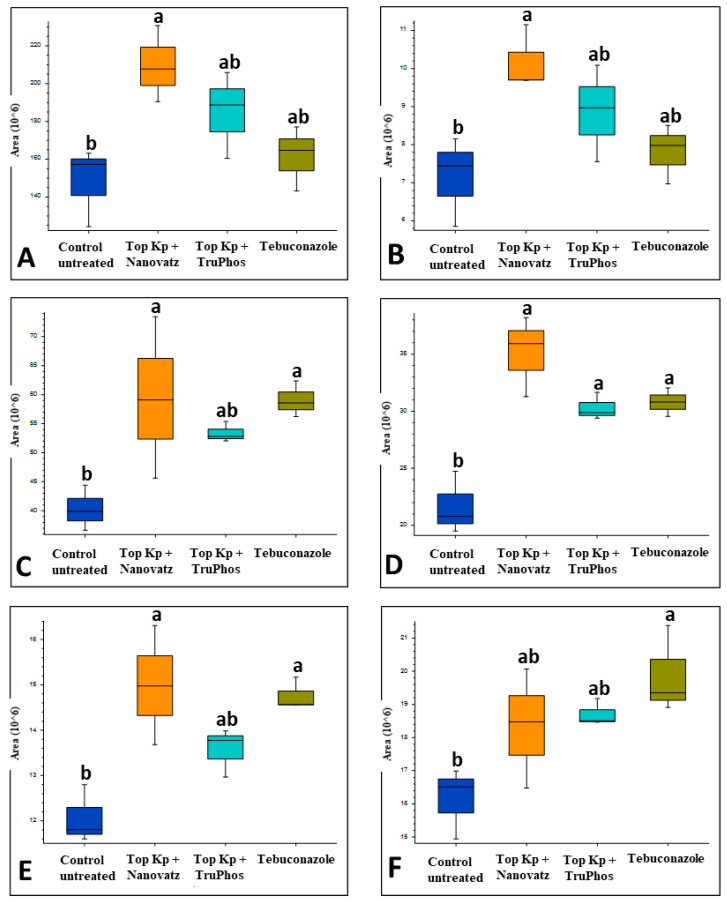
Relative amounts (peak area) of metabolites that were up-produced in grape skins of cv. Carignan vines treated with six foliar spays (starting at April 6 until June 8) of the fungicide Folicur (tebuconazole) 0.02%, or the fertilizers Top KP+ 1% plus Nanovatz 0.1%, Top KP+ 1% plus TruPhos 0.25%, compared to control untreated vines. Analysis was conducted using LC–MS/MS. (**A**) Trans-3-Indoleacrylic acid, (**B**) Indole, (**C**) Phytosphingosine, (**D**) 6-hydroxysphing-4E-enine, (**E**) Limocitrin, (**F**) (±)-(2E)-Abscisic acid. Different letters represent significant differences (*p* < 0.05, Tukey-Kramer HSD test).

PCA analysis of 205 compounds identified by LC-MS/MS in cv. Carignan grape skin tissues also revealed that compound composition in untreated grapes was significantly different (*p* < 0.05) from those in grapes treated by the fungicide Folicur, Top KP+ plus Nanovatz, or Top KP+ plus TruPhos (Figure 6). Nineteen metabolites were significantly upregulated in at least one of the treatments of sprayed grapes (fertilizers or fungicide) compared to untreated control grapes (Figure 7, Table S3). Among 19 metabolites that were significantly upregulated, six were annotated (Table 5). The amount of the organic acid trans-3-Indoleacrylic acid and the aromatic compound Indole was significantly higher (*p* < 0.04) in grapes treated with Top KP+ plus Nanovatz than untreated grapes (Figure 8A,B). The amount of the sphingoid bases Phytosphingosine and 6-hydroxysphing-4E-enine was significantly higher (*p* < 0.05 and *p* < 0.006, respectively) in grapes treated with Top KP+ plus Nanovatz, or by the fungicide Folicur, while Top KP+ plus TruPhos was significant only in 6-hydroxysphing-4E-enine, compared to the untreated grapes (Figure 8C,D). The concentration of the flavonol Limocitrin was also significantly higher (*p* < 0.01) in grapes treated with Top KP+ plus Nanovatz, or by Folicur (Figure 8E). Treatment of Folicur enhanced production of the phytohormone Abscisic acid compared to untreated grapes (Figure 8F).

### 2.4. Activity of Macro and Micronutrients In Vitro and in Planta

#### 2.4.1. Prophylactic Efficacy of Macro and Micronutrients against Powdery Mildew

**Figure 9:**
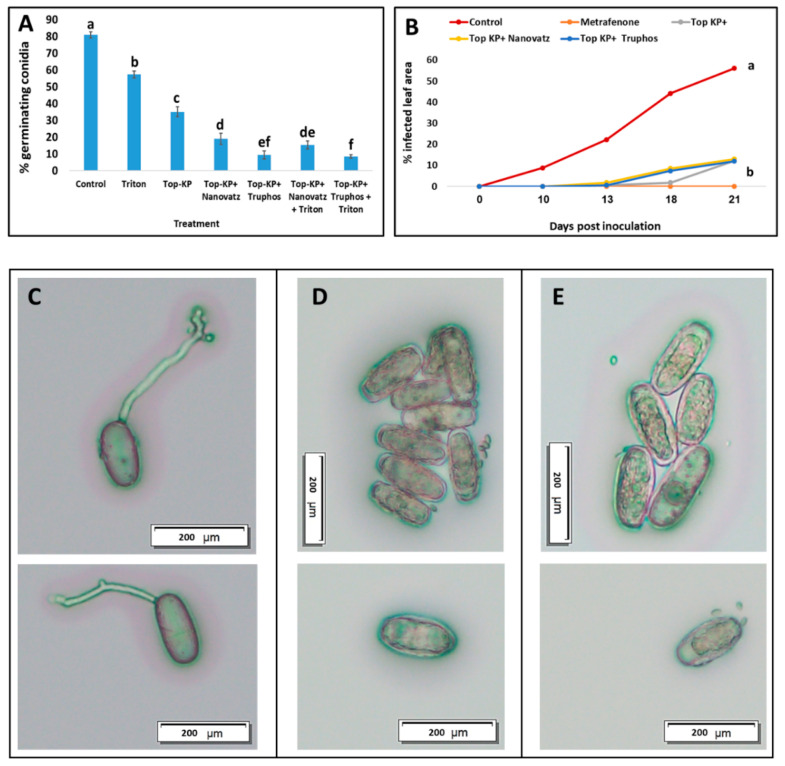
(**A**) The effect of Top KP+ 1% plus TruPhos 0.25% or Nanovatz 0.1% on conidial germination of *E. necator* in vitro. Bars represent the standard error of the mean. (**B**) Prophylactic activity of Top KP+ 1% plus TruPhos 0.25% or Nanovatz 0.1% in comparison to Vivando (metrafenone) 0.03% fungicide against grape powdery mildew on young plants. The percentage of infected leaf area on five leaves of each treated plant was recorded at various time intervals after foliar spray. Different letters indicate significant differences (*p* < 0.05) according to the Fisher’s LSD K-ratio *t*-test. (**C**) Germinating conidia on water agar. (**D**) Inhibition of conidial germination, and disrupted conidia with shrunken intra-cellular content on water agar embedded with Top KP+ 1% plus TruPhos 0.25%. (**E**) Inhibition of conidial germination, and disrupted conidia with shrunken intra-cellular content on water agar embedded with Top KP+ 1% plus Nanovatz 0.1%. Bar = 200 µm.

In control plates, conidial germination reached a mean of 81%. While Top KP+ alone inhibited conidial germination in vitro by only 56%, the mixtures of Top KP+ plus Nanovatz, or TruPhos inhibited conidial germination by 76% and 86%, respectively, relative to control (Figure 9A). The surfactant Triton X100 inhibited conidial germination by only 28%, and adding it to the nutrient mixtures increased their efficacy by 3–5% (Figure 9A). In potted grape plants, Top KP+ and Top KP+ plus TruPhos, or Nanovatz inhibited powdery mildew development by 80% compared to control untreated plants, as recorded 21 days post inoculation, and were not significantly different from the fungicide Vivando (metrafenone) (Figure 9B). Microscopical examination of conidia on WA showed plasmolyzed conidia with shrunken intra-cellular content in treated samples, as compared to normal cytoplasm in the control conidia (Figure 9C–E).

#### 2.4.2. Direct Effect of Macro and Micronutrients on Powdery Mildew

**Figure 10:**
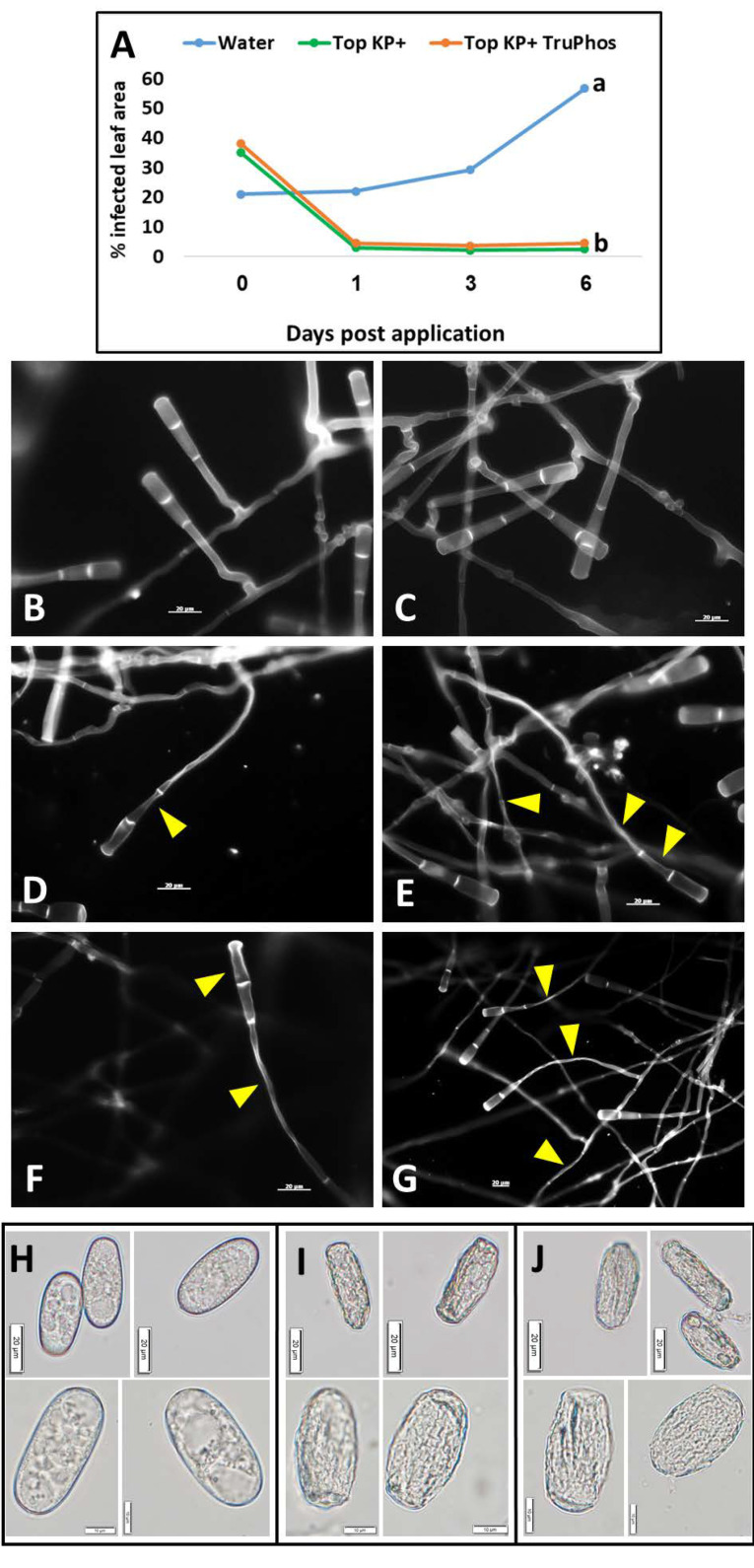
(**A**) Curative effect on existing powdery mildew colonies on young grape plants by a single application of Top KP+ 1%, and the mixture Top KP+ 1% plus TruPhos 0.25%. Different letters indicate significant differences (*p* < 0.05) according to the Fisher’s LSD K-ratio *t*-test; (**B**–**G**) Epifluorescent micrographs of hyphae and conidia of *E. necator* from curative experiment: (**B**–**C**) Normal conidiophores and hyphae with smooth hyphal walls from control water treated colony. (**D**–**E**) Disrupted conidiophores (marked by arrows), and shrinkage and disruption of hyphae (marked by arrows) on infected leaves exposed to a single application of Top KP+ 1% plus Nanovatz 0.1%. (**F**–**G**) Disrupted conidia and conidiophores, and deformed hyphal cells (marked by arrows) on infected leaves exposed to a single application of Top KP+ 1% plus TruPhos 0.25%. Samples for microscopy were taken 24 h post-application. Bar = 20 µm. (**H**–**J**) Light microscopy of *E. necator* conidia: (**H**) Normal conidia taken from colonies sprayed with water. (**I**) Deformed and shrunken conidia taken from colonies exposed to a single application of Top KP+ 1% plus Nanovatz 0.1%. (**J**) Deformed and shrunken conidia taken from colonies exposed to a single application of Top KP+ 1% plus TruPhos 0.25%. Samples for microscopy were taken 24 h post-application. Bar = 20 µm (×100 magnification), or 10 µm (×400 magnification).

A single spray of Top KP+ or Top KP+ plus TruPhos applied to the upper leaf surface of infected plants stopped further colonization of the leaves with powdery mildew. The spray disrupted the colonies and prevented their further spread. At six days after spray, control plants treated with water showed 55% coverage with powdery mildew colonies, as against 3–5% coverage in fertilizers-treated plants (Figure 10A). Microscopic examination under UV light of infected leaf discs taken from control plants revealed normal conidia and condiophores (Figure 10B,C), but morphologically disrupted fungal hyphae and conidiophores in discs taken from treated plants (Figure 10D–G). Light microscopy observations revealed normal conidia in untreated control leaves (Figure 10H) but plasmolyzed conidia in treated leaves (Figure 10I,J).

## 3. Discussion

The need to prevent the negative impact of synthetic pesticides on human health and the environment stimulated the search for innovative tools and methods for sustainable pest management [1]. In the present study, we demonstrate that the simple compound Top KP+, which contains Potassium (K) and Phosphorus (P), or Top KP+ combined with ready mixtures of micronutrients (Zinc (Zn), Boron (B), Copper (Cu), Iron (Fe), Manganese (Mn) and Molybdenum (Mo)) were highly effective as synthetic fungicides in controlling powdery mildew on foliage and fruits of field-grown susceptible grapevines.

The literature teaches that the use of inorganic salts for fungal disease control is a well-known concept in crop protection. Early reports date back to Prévost (1807) [67], who applied copper sulphate (CuSO_4_) against the bunt of wheat caused by *Tilletia caries* [68]. Bordeaux mixture, made of CuSO_4_ and Ca(OH)_2_, is one of the earliest fungicides used for disease control worldwide [69]. Sodium bicarbonate effectively controlled powdery mildew in various crops [11,12,70]. Silicon in combination with potassium and phosphorus reduced powdery mildew severity in cucurbits [71]. Foliar sprays of phosphates were effective against powdery mildews in trees, including mango and nectarine [19], apple [13], and wine grapes [17,19,72]. The effects of combinations of macro and micronutrients was reported in several studies. Oprica et al. [73] reported that the foliar application of a mixture of N, P, K, Fe, Cu, and Mn enhanced nutrient content in leaves and seeds of maize and sunflower, and increased yield by 50% compared to the basal application of NPK alone. Younis et al. [74] showed that the combination of macro and micronutrients improved the yield and quality of roses, compared to macronutrients alone. Awan et al. [75] demonstrated the synergistic effect of zinc with other nutrients in managing early blight in tomato. However, the effect of combining macro and micronutrients on grape powdery mildew was never reported. Here, we show that (i) foliar applications of macro-nutrients were effective in controlling grape powdery mildew, and (ii) foliar applications of the mixture of macro-nutrients with micronutrients were more effective compared to their single application.

In order to reduce the buildup of fungicide resistance, it is recommended to use fungicide mixtures or fungicide alternations [76]. The integration of multi-site fungicides [76] or fertilizers into the management program may further reduce the buildup of resistance. We demonstrate here that the integration of Top KP+ plus TruPhos or Nanovatz in the spraying program, in alternation with systemic fungicides, was as effective as systemic fungicides all along the season. Furthermore, applying those nutrients in a tank mixture with a fungicide improved disease control.

Foliar applications of B and Zn to maintain adequate micronutrient concentrations and to prevent inhibition of reproductive growth is a common vineyard management practice [77,78]. Our results showed that integrating nutrient mixtures in alternation with fungicides, or in mixtures with fungicides in the spray program against grape powdery mildew, improved disease control. The fact that in most experiments TruPhos (which contain more micronutrients than Nanovatz) was as effective as Nanovatz may indicate that the most essential elements for disease control are Zn and B.

Fungicides are combined in mixtures to expand their spectrum of activity, to prolong their persistence, and to improve disease control by exploiting synergistic interactions between their components [79,80]. Synergy, which frequently occurs between fungicides in mixtures, may involve antifungal compounds of differing natures and sources, differing or identical modes of action, or differing formulations [79]. The enhanced protection induced by Top KP+ plus, TruPhos, and Nanovatz when mixed with the sterol inhibitor fungicide Folicur (tebuconazole) compared to Folicur alone indicates a synergistic interaction between the fertilizers and the fungicide. Further research is required to confirm this.

Mineral nutrition may affect plant resistance or susceptibility to disease [81]. In general, P and K tend to improve plant health, while in most cases, N increases plant susceptibility to disease [13]. Top KP+ and TruPhos used in this study were chosen because they contain mainly P and K with low N content. Microelements may also play an important role in plant health by affecting their susceptibility to pathogens [82]. Mechanisms leading to such nutrient-induced changes in disease resistance/susceptibility are complex and include direct effects of mineral nutrients on the pathogen, on plant resistance mechanisms, and on plant growth and development [83]. The effect of macro and micronutrients used in this study on plant growth was not tested, but the high efficacy of the nutrient mixtures against grape powdery mildew in the field further calls to study the effects of the nutrients on vine growth, productivity, and quality.

The modes of activity of foliar-applied nutrients in controlling fungal diseases have been reported by Reuveni et al. [20,84]. They demonstrated prophylactic control activity of phosphate salts against *Sphaerotheca fuliginea* in cucumbers and *Leveillula taurica* in peppers, and curative activity in cucumbers when applied onto existing mildew colonies [18]. Phosphonic acid (H_3_PO_3_) reduced the incidence of grape downy mildew and the level of sporulation of *Plasmopara viticola* when applied after infection [85].

However, the exact mode of action of foliar-applied macro and micronutrients in controlling *E. necator* on grapevines has not been yet clearly elucidated. The present study demonstrates that foliar nutrient sprays exhibit both prophylactic and curative activity against *E. necator* in potted grape plants. While the surfactant alone showed very limited inhibition of conidial germination in vitro, and macronutrients alone (Top KP+) exhibited partial inhibition, macro plus micronutrient mixtures further inhibited conidial germination. They caused disruption and shrinkage of hyphae, conidiophores, and conidia. Such deformations probably result from the osmotic effect of the salts, which disrupt the membrane integrity of fungal cells, causing plasmolysis and leakage of cell content [18]. Besides their direct toxicity to fungal structures, nutrient mixtures may also induce local and systemic resistance in grapevines against *E. necator*, as was demonstrated by phosphate salts and micronutrients in cucumber plants infected by *Sphaerotheca fuliginea*, and maize plants effected by *Puccinia sorghi* or *Exserohilum turcicum* [20,86,87,88].

Nutrients treatment did not improve nor damage the oenological parameters Brix and pH. This is in line with the work of Reuveni and Reuveni [89]. Foliar applications of micro and macronutrients slightly increased berry weight, but not significantly as reported by Reuveni and Reuveni [89], who showed an increase in the weight of grape clusters treated with phosphate.

Comparing the metabolites profiles of severely infected untreated berries to that of relatively healthy berries sprayed with macro and micronutrients or a fungicide showed a significantly upregulated production of several metabolites in the treated berries. Riesling berries treated with nutrients or fungicide produced significantly higher amounts of the dipeptides Leu-Arg, dihydroxy-phenylalanine, Boc-Ser-OH, Boc-Gln-OH, and L-gamma-glutamyl-L-leucine compared to untreated berries. Numerous compounds that confer resistance to fungal pathogens were identified in plants, among them proteins and peptides with anti-microbial activity [36,37]. Anti-microbial peptides, or host defense peptides, are important components of innate immune systems [38]. The higher amount of dipeptides in berries treated with nutrients or fungicide may be related to the suppression of the pathogen by these antibiotic metabolites. Dipeptides have also been reported to impart a range of tastes, including umami and sweetness, and have previously been detected in wines, considered a positive contribution to wine quality [40,41]. The contribution of dipeptides to the antioxidant stability of wines has also been hypothesized [39]. It seems that the high content of dipeptides in berries treated with nutrients or fungicide has an added value to wine quality, besides their disease control aspect. Berries treated with nutrients or fungicide also contained a higher amount of pheophorbide A. Pheophorbide is a plant-derived chlorophyll metabolite associated with several bioactivities (i.e., antiviral, anti-inflammatory, antioxidant, immunostimulatory, anti-parasitic) [42,43]. Treated cv. Carignan berries also produced more flavonols known for their antioxidant activity [55,56]. Antioxidant compounds are believed to be responsible for the health effects of moderate wine consumption because they can quench free radicals, and therefore minimize oxidative stress damage [90]. Two additional metabolites, 6-tuliposide B and Nocardicin, were upregulated in treated berries as well. They are known for their potent antimicrobial activity [44,45,47]. Further, 6-tuliposide B also showed an antifungal inhibitory effect against *Pythium ultimum*, *Rhizoctonia solani*, and *Fusarium* spp. [46].

The two metabolites, cis-resveratrol (stilbenoid) and Catechin 3-O-gallate (flavan-3-ol), were significantly upregulated in the severely mildew infected berries of the control untreated grapes. Grape stilbenes are phytoalexins produced by the plant in response to abiotic and biotic stresses, including fungal infection [91,92]. Resveratrol is a well-known bioactive stilbene [93]. Romero-Pérez et al. [94] showed that the content of trans-resveratrol was considerably increased in grape berries infected with powdery mildew, and the degree of infection was positively related to their stilbene content. Naturally mildew-infected grape berries and leaves exhibited an increase in cis-resveratrol and catechin [26,95].

Treated cv. Carignan berries contained more organic compounds such as indoles. Dohgo et al. [50] reported on the inhibitory effect of indole on the haustorial formation of barley powdery mildew. Indole primes defense signaling and increases herbivore resistance in tea plants [51]. It may also be related to defense signaling against mildew attack in grapes. The content of sphingoid bases was also upregulated in treated berries. Sphingoids exhibited antifungal effects against *Fusarium oxysporum* in watermelon [52] and induces systemic acquired resistance against *Phytophthora parasitica* var. *nicotianae* [53]. The phytohormone Abscisic acid, a regulator in plant biotic defense responses [57,58], was also upregulated. The role of these antifungal metabolites against grape powdery mildew should be further examined.

A possible indirect mode of activity of the fertilizers derived from changes in berry metabolic profile was demonstrated by comparing the metabolites composition of relatively healthy grapes treated with the fungicide Folicur (tebuconazole) with those treated with the macro and micronutrients. Results revealed several metabolites that were significantly upregulated in grapes of cv. Riesling treated with the fertilizers. Macro and micronutrient sprays enhanced the production of antifungal compounds, such as the non-protein amino acid L-homoarginine. L-homoarginine is an alkaline phosphatase inhibitor that interrupts phosphorus metabolism in fungal hyphae [59]. It inhibited the growth of *Torulopsis utilis* and *Neurospora crassa* [60,61,96]. Simola and Lonnrothl [62] showed that the amino acid composition of a plant influences the growth of parasitic fungi and that some of the non-protein amino acids such as homoarginine may protect the plant against infection. The effect of fungicide or alternative treatments against grape downy mildew (caused by *Plasmopara viticola*) on grape amino acids was also reported [97]. Further molecular and biological studies are needed to shed light on the mechanism of action of these metabolites and their activities. In addition, the effect of foliar fertilizers application on the berries’ skin microbiome and its relation to berries metabolomics should be further investigated.

Two carbohydrates, 2-Deoxy-2-(methacryloylamino)-D-glucopyranose and the ester 6-O-Acetyl-D-glucose, were upregulated following nutrient treatment. Carbohydrates exert important biochemical functions (energy supply, molecular recognition processes, structural roles, cell-surface functions) and are precursors of various molecules, particularly bioactive compounds, such as oligosaccharides, glycol-conjugates, and nucleosides [63].

Macro and micronutrient treatment also significantly upregulated three organic acids. Organic acids play an important role in determining wine quality, and their organoleptic properties affect wine tests. Their salts also act as buffers, thus ensuring that the wine maintains a relatively low pH to protect it against bacterial attack and subsequent spoilage. These acids also help conserve wine color and influence esterification with a consequent impact on the bouquet [65]. The taste profile of wine accounts for several orosensory qualities, e.g., sourness, sweetness, bitterness, and astringency. Some of the perceived sourness in wine was imparted by the upregulated organic acids D-galacturonic acid, succinic acid, and glutaric acid [66]. Nutrient sprays did not significantly enhance the content of phenolic compounds, including anthocyanins, flavanols, and stilbenes, in grape tissues following macro or micronutrient applications.

Macro and micronutrient sprays enhanced the production of antifungal and antioxidant metabolites more than fungicide sprays. Since these compounds are also involved in induced resistance against pathogens, the remarkable increase in these metabolites compared with untreated or fungicide-treated berries suggests a possible indirect activity against powdery mildew and the direct inhibiting effect of the nutrients on the pathogen. Micronutrients, such as zinc and copper, are known to be involved in enzyme activities that play a role in plant induce resistance, such as polyphenol oxidase activity [98] and superoxide production [99]. This, together with the additive advantage to wine quality, should encourage integrating nutrient sprays in disease management programs.

In summary, our field and laboratory trials showed that foliar sprays of fertilizer mixtures consisting of macro (here Top KP+) and micronutrients (mainly containing Zn and B) are highly effective, like fungicides, in controlling powdery mildew on both leaves and fruit clusters of grapevines. Possible mechanisms responsible for fertilizers’ efficacy include the preventive inhibition of infection and colonization of the leaves and berries with powdery mildew due to the inhibition of conidia germination, plasmolysis of fungal mycelia and conidia, and upregulated production of antifungal compounds that might induce systemic resistance. The rapid absorption of fertilizers by the plant tissues, their mobility within tissues, low animal toxicity, environmental safety, and nutritional value make them suitable for disease control. The combination of the fungicide Folicur with the fertilizers improved efficacy compared to Folicur alone. Integrated pest management (IPM) programs that combine nutrients and fungicides at proper timing may suppress disease and reduce the buildup of fungicide resistance. They will also protect berries against disease and alter their metabolic profile to improve wine quality.

## 4. Materials and Methods

### 4.1. Nutrients and Fungicides

The following commercial fertilizers were examined: Top KP+ (NutriAg, Toronto, ON, Canada) containing N:P:K (1:50:33), Nanovatz (also called ZincMax) (NutriAg, Toronto, ON, Canada) containing 102 g/L chelated Zinc (Zn) and 5 g/L Boron (B), and TruPhos Platinum (NutriAg, Toronto, ON, Canada) containing N:P:K (5:18:2) and Magnesium (Mg) 5 g/L, Boron (B) 1 g/L, Cobalt (Co) 0.5 g/L, Copper (Cu) 1 g/L, Iron (Fe) 1 g/L, Manganese (Mn) 0.5 g/L, Molybdenum (Mo) 0.5 g/L, and Zinc (Zn) 8 g/L. Top KP+ was applied alone or as a tank mix with Nanovatz, or with TruPhos Platinum. The nonionic surfactant Triton X100 (ADAMA-Agan, Ashdod, Israel) was added to all fertilizers at 0.025% (*v*/*v*) before application.

Four fungicides were used in field and laboratory studies for comparison purposes: Abir 250SC (Dow AgroSciences, IN, USA) containing quinoxyfen (FRAC code 13), Heliosulfur 700SC (Action Pin, Castets, France) containing mineral sulfur (FRAC code M02), Folicur 250EC (Bayer CropScience Ltd., Monheim, Germany) containing tebuconazole (FRAC code 3), and Vivando 500SC (BASF, Ludwigshafen, Germany) containing metrafenone (FRAC code 50).

### 4.2. Experimental Vineyards and Location

Five field trials were conducted from 2018 to 2020 with the powdery mildew susceptible grape cultivars: ‘Cabernet Sauvignon’ (trial 1; 2018), ‘Chardonnay’ (trials 2 and 3; 2019 ‘Riesling’ (trial 4; 2020), and ‘Carignan’ (trial 5; 2020). Vineyards were located in the Golan, Upper Galilee, and Judean foothills regions of Israel (Table 6). Field trails 1–3 and field trials 4 and 5 represent different research questions. The efficacy of the nutrients in field trials 1–3 were compared to highly effective synthetic reference fungicide (Abir) and to an effective commonly used organic product (Heliosulfur). In trials 4 and 5, the efficacy of mixtures of nutrient plus fungicide was tested compared to nutrients or fungicide alone. Therefore, in order to demonstrate the enhanced effect of adding nutrients to the mixture, in trials 4–5, we used Folicur, which is considered a fungicide with moderate effect against grape powdery mildew in Israel. In addition, resistant isolates of *E. necator* to Folicur were reported in Israel [100], therefore emphasizing the need for additional control measures against grape powdery mildew.

### 4.3. Nutrient and Fungicide Applications

Fungicides and nutrients were applied to vineyards at recommended concentrations (Table 6), according to phenological stages and a Decision Support System (DSS) “Eshkol” (“Cluster”) [101]. Applications were based on three phenological periods. The first period included one or two prophylactic sprays at the early season (from the first leaf unfolded stage (BBCH-11) to start flowering (BBCH-57)) in cv. Carignan, or application before any rain event in the other cultivars. The second period included 2–3 sprays to all cultivars from bloom (BBCH-60) to berries at “beginning to touch” (BBCH-77) phenological stage [101]. In the third application period, sprays were given according to disease monitoring on berries. Details on each trial are summarized in Table 6.

Fungicides were sprayed with a Turbo 400 (100-L, 1400 kPa) gun sprayer (Degania Sprayers, Degania, Israel) at spray volumes of 800–1000 L ha^−1^, according to the size of the vines, at time intervals specified for each trial on phenological and primary infection basis. Unsprayed vines served as control. In all trials vines were spur pruned and vertical shoot position (VSP) trained. Spacing between rows and vines was 4 × 2 m or 3 × 1.5 m. Treatments in all field trials were arranged in a randomized complete block design. Plots consisting of 6–8 adjacent vines were replicated four times. Disease evaluations were conducted on 4–5 central vines to ensure prevention of drift from the adjacent treatment. Unsprayed buffer rows ensured the prevention of drift from the adjacent commercial plots. Methods of fertilization, irrigation, and other cultural practices were as recommended to growers in the region and equal for all treatments. Basal leaf removal (all leaves up to the first cluster) was conducted on both sides of the vines four weeks after bloom.

### 4.4. Recording Powdery Mildew on Leaves and Fruit Clusters

Leaves and clusters that were naturally infected with powdery mildew were rated once or twice after the last application of fungicides and nutrients. Twenty-five leaves or clusters were randomly selected from each side of each row of the four center vines in each replicate plot (50 leaves/clusters per replicate, 200 leaves/clusters per treatment) and rated for disease severity according to the percentage of infected leaf or cluster area (severity). The percentage of leaves/clusters infected with powdery mildew (incidence) was also determined.

### 4.5. Effects of Nutrients and Fungicide on Must Oenological Parameters and Berry Weight

The effect of Top KP+ 1% plus Nanovatz 0.1%, Top KP+ 1% plus TruPhos 0.25%, and Folicur 0.02% on must oenological parameters, including pH and sugars content (Brix), was determined in two vineyards. Berries were collected in 2020 from vines of two field trials: (i) Berries of white grape cv. Riesling in Eshtaol vineyard trial (Trial 4) were collected on 9 August 2020, which was 17 days before commercial harvest. (ii) Berries of red grape cv. Carignan in Mazkeret-Batya vineyard trial (Trial 5) were collected on 30 August 2020, which was 3 days before commercial harvest. Five berries were randomly collected from the upper and middle part of each of 20 clusters (10 clusters from each side) in each replicate plot (100 berries per replicate, 400 berries per treatment). Berries were macerated for the must quality. The pH of must was measured using a pH meter (PH700 Benchtop, Apera Instruments, OH, USA) and Brix was measured with the aid of a refractometer (PAL-1, ATAGO, Japan). In addition, in Mazkeret-Batya vineyard, the weight of each of 50 berries of each replicate (200 berries per treatment) was determined before must preparation.

### 4.6. Effect of Nutrients on Berry Metabolites

#### 4.6.1. Sample Collection and Preparation

In order to examine the possible effects of the foliar application of fertilizers on berry metabolomics related to antifungal activity or improvement of wine quality, additional collection for metabolomics analysis was conducted in parallel to the collection of oenological parameters. Berries from the two field trials in Eshtaol vineyard (cv. Riesling) and in Mazkeret-Batya vineyard (cv. Carignan) were collected in 2020 from vines treated with Top KP+ 1% plus Nanovatz 0.1%, Top KP+ 1% plus TruPhos 0.25%, Folicur (tebuconazole) 0.02%, and untreated control vines as described above. Berries were placed in plastic boxes, stored in a cooler on ice, brought to the laboratory and immediately frozen (−20 °C) until analysis. The skin was manually separated from frozen berries, and one gram of skin tissue was ground to powder under liquid nitrogen. The frozen powder was lyophilized for 24 h and thereafter extracted with 5 mL methanol containing 0.1% formic acid. The samples were homogenized for 1 min (2000 rpm) and centrifuged (4200 rpm, 10 min, 4 °C). The supernatant was filtered through 0.22-µm PTFE syringe filters (Membrane Solutions, Plano, TX, USA). The extraction procedure was repeated twice, and supernatants were combined. The extract was diluted 10-fold with Methanol into a HPLC vial and injected into LC-MS (liquid chromatography–mass spectrometry). Pooled samples were made by mixing all the samples into one HPLC vial for quality control and MS^2^ fragmentation.

#### 4.6.2. HPLC Analysis

Samples were analyzed by injecting 5 μL of the extract into a UHPLC (ultra-high-performance liquid chromatography) connected to a photodiode array detector (Dionex Ultimate 3000, Dionex, Idstein, Germany), with a reverse-phase column (ACE C18, 100 × 3.0 mm, 1.7 μm, Avantor, PA, USA). The mobile phase consisted of solvent A (Double distilled water containing 0.1% formic acid) and solvent B (acetonitrile containing 0.1% formic acid). The gradient started with 5% B and kept isocratic for 2 min, then increased to 98% B for 23 min and kept isocratic at 98% B for 5 min. Phase B returned to 5% for 2 min and the column was allowed to equilibrate at 5% B for 5 min before the next injection. The flow rate was 0.4 mL/min.

#### 4.6.3. LC–MS/MS Analysis

Liquid chromatography linked to tandem mass spectrometry (LC–MS/MS) analysis was performed with Heated Electrospray Ionization (HESI-II) source connected to a Q Exactive™ Plus Hybrid Quadrupole-Orbitrap™ Mass Spectrometer (Thermo Fisher Scientific, MA, USA). Electrospray ionization (ESI) capillary voltage was set to 3000 V, capillary temperature to 350 °C, gas temperature to 350 °C and gas flow to 35 mL/min. The mass spectra (m/z 100–1500) were acquired in negative and positive-ion mode with high resolution (full width at half maximum (FWHM) = 70,000). For MS^2^ fragmentation analysis, collision energy was set to 15, 50, and 100 EV.

#### 4.6.4. LC–MS/MS Data Preprocessing

Peak determination and peak area integration were performed with Compound Discoverer 3.1 (Thermo Xcalibur, Austin, TX, USA, Version 3.1.0.305) and with QuanBrowser (Thermo Xcalibur, USA, Version 4.1.31.9). Auto-integration was inspected manually and corrected when necessary. Compounds were identified based on mzCloud database using MS^2^ data and ChemSpider database using HRMS (High Resolution Mass Spectra). A pooled sample was injected once after each 6 samples for normalization of peak area. RSD (relative standard deviation) of the pooled sample was calculated for each compound and area correction was made automatically using CD 3.1 algorithm.

### 4.7. Mode of Activity of Macro and Micronutrients In Vitro and in Planta

#### 4.7.1. Plants

Grapevine seedlings (*Vitis vinifera*) cv. ‘Chardonnay’ were grown in 5 × 5 cm pots containing garden planting soil mixture in a growth chamber at 25 °C, with a light intensity of 100–120 µE·m^−2^·s^−1^ with 14 h photoperiod. Plants at 4–6 leaf stage were used for experiments.

#### 4.7.2. Inoculum Production and Inoculation

An isolate of *E. necator*, obtained from an infected vineyard in the Golan region, Israel (isolate 146, [102]), was maintained on detached young leaves of *V. vinifera* cv. Cabernet Sauvignon in a growth chamber at 23 °C, 14-h light photoperiod under fluorescent light of 100 µmole·m^−2^·s^−1^. Inoculum was obtained from freshly sporulating leaves 10–12 days after inoculation. Aqueous conidial suspension was prepared by gently brushing conidia from the mildew colonies into 1.5-mL microcentrifuge tubes containing sterile distilled water. The conidial suspension was adjusted to 1 × 10^4^ conidia·mL^−1^, and each plant was spray-inoculated with ~500 μL conidial suspension. The inoculated plants were placed in complete randomization in a growth chamber at 23 °C, 14-h light photoperiod under fluorescent light of 100 µmole·m^−2^·s^−1^.

#### 4.7.3. Inhibitory Effect of Macro and Micronutrients on Conidial Germination In Vitro

Conidia of *E. necator* from 10-days old colonies (isolate 146, [102]) grown on cv. Cabernet Sauvignon detached leaves were gently brushed above 9-cm petri dishes containing 2% water agar (WA) amended with 1% Top KP+ (containing 0.1, 5, and 3.3 g/L N:P:K respectively), 1% Top KP+ plus 0.1% Nanovatz (containing 0.102 and 0.005 g/L Zn and B respectively), 1% Top KP+ plus 0.25% TruPhos (containing 0.125, 0.45, and 0.05 g/L N:P:K respectively, and 0.02, 0.0025, 0.0125, 0.00125, 0.0025, 0.0025, 0.00125, and 0.00125 g/L Zn, B, Mg, Co, Cu, Fe, Mn, and Mo respectively), 1% Top KP+ plus 0.1% Nanovatz plus 0.025% Triton, 1% Top KP+ plus 0.25% TruPhos plus 0.025% Triton, or only 0.025% Triton. Petri dishes containing 2% WA alone were used as controls. Dishes were incubated at 24 °C in the dark for 24 h. The number of germinating conidia was counted in 3 fields of view of each dish under the microscope at 100× magnification (approximately 25 conidia per field, total of 225 conidia per treatment). Percent of germinated conidia was determined. Three petri dishes were used for each treatment, and each experiment was conducted twice.

#### 4.7.4. Prophylactic Efficacy of Macro and Micronutrients against Powdery Mildew

The upper and lower leaf surfaces of 6-leaf plants (four plants per treatment) of cv. ‘Chardonnay’ were sprayed to run off (by using a hand sprayer) with freshly prepared aqueous solutions of Top KP+ (1% *w*/*v*), Top KP+ (1% *w*/*v*) plus TruPhos (0.25% *v*/*v*) or plus Nanovatz (0.1% *v*/*v*), or the fungicide Vivando (metrafenone) (0.03% *v*/*v*). Control plants were sprayed with water. Triton X100 (0.025% *v*/*v*) was added to all fertilizers or water before application. Spray droplets were allowed to dry, and plants were kept in a growth chamber (23 °C, 14-h light photoperiod). One day later plants were inoculated by spraying 0.5 mL of a conidial suspension containing 1 × 10^4^ conidia/mL per plant and returned to the growth chamber for disease development. On each leaf, the proportion of leaf area infected with powdery mildew was evaluated at 10, 13, 18, and 21 days after inoculation.

#### 4.7.5. Direct Effect of Macro and Micronutrients on Powdery Mildew

Six-leaf plants cv. ‘Chardonnay’ were spray-inoculated with *E. necator* conidial suspension containing 1 × 10^4^ conidia/mL. Thirteen days after inoculation, when powdery mildew colonies were fully developed on the foliage, the percent infected leaf area of each leaf was evaluated on each of the four plants. Plants were sprayed on their upper surfaces with 0.5 mL of Top KP+ (1% *w*/*v*), Top KP+ (1% *w*/*v*) plus TruPhos (0.25% *v*/*v*), or water for each plant. Triton X100 (0.025% *v*/*v*) was added to all fertilizers before application. After spraying, plants were arranged in a complete randomized block design and incubated in a growth chamber (23 °C, 14-h light photoperiod). The percentage of infected leaf area on each leaf was visually estimated at 1, 3, and 6 days after spray application.

#### 4.7.6. Microscopic Examination of Suppression Activity

In order to examine the curative suppression activity of the nutrients on the fungus organs, microscopic observations were undertaken. Six-leaf plants cv. ‘Chardonnay’ were spray inoculated with conidial suspension of *E. necator* as described above. Ten days after inoculation, when powdery mildew colonies were fully developed, each leaf was sprayed with Top KP+ (1% *w*/*v*), Top KP+ (1% *w*/*v*) plus TruPhos (0.25% *v*/*v*) or plus Nanovatz (0.1% *v*/*v*), or water as described above. Twenty-four hours after spraying, conidia were gently removed from the treated leaves with the aid of a biological needle and examined under a microscope (Olympus- BX43, Japan) under bright-field illumination (×100 and ×400 magnifications). In order to further examine the effect of the nutrients on hyphae and conidiophores using fluorescent microscopy, the procedures described by Cohen et al. [103] were employed. Leaf discs 12 mm in diameter were removed from the test leaves 24 h after treatment with fertilizers or water. Leaf discs were placed in 2 mL ethanol and boiled for 15 min to clarify. The ethanol was discarded, and 5 mL of 0.01% aniline blue solution (in 0.1 M dibasic-potassium phosphate pH 8.9 buffer) were added. The vials containing the leaf discs were kept at 4 °C overnight. The leaf discs were then removed, placed on glass slides, lower surface upper-most, treated with 10 μL of 0.05% calcofluor solution (Sigma), covered with a coverslip, and examined with the aid of an upright fluorescence microscope (Olympus-BX43, Japan).

### 4.8. Data Analysis

Each laboratory experiment was conducted at least twice. All data were analyzed with the JMP statistics package version 14.1 (SAS, Cary, NC, USA). For experiments with plants in the growth chamber or field experiments, one-way analysis of variance (ANOVA) was applied to percent infected leaves or clusters (incidence) and to arcsin transformed data of the percent infected leaf or cluster area parameters (severity), in order to achieve a normal distribution. Data in tables are reported in untransformed units. Fisher’s LSD K-ratio *t*-test was applied to determine whether differences between treatments were significant at α = 0.05. Hierarchical cluster analysis of metabolites was conducted using MetaboAnalyst (version 4.0) [104]. The Tukey–Kramer HSD test was applied to determine whether differences between metabolites quantity in grapes under each treatment were significant at α = 0.05. Principal components analysis (PCA) analysis for 840 compounds identified by LC-MS/MS in grape skin tissues treated by the fungicide Folicur (tebuconazole) 0.02%, Top KP+ 1% plus Nanovatz 0.1%, or Top KP+ 1% plus TruPhos 0.25% and untreated grapes was conducted using the software Compound Discoverer 3.1 (Thermo Xcalibur, USA, Version 3.1.0.305).

## Figures and Tables

**Table 1:** Efficacy of nutrients and fungicides in controlling powdery mildew on the clusters of *Vitis vinifera* cv. Cabernet Sauvignon at Merom-Golan vineyard (trial 1), 2018.

Treatment ^1^	6 Days Post Sixth Application	
	Incidence (Clusters)	Severity (Clusters)
Control	100 a ^4^	86.4 a
Top KP+ (1%) + Nanovatz (0.1%)	95 ab	4.5 b
Top KP+ (1%) + TruPhos (0.25%)	94.5 ab	2.8 c
Abir 250SC ^2^ (0.02%)	79.5 b	1.6 c
Heliosulfur 700SC ^3^ (0.5%)	88.5 ab	2.2 c

^1^ For each treatment, six foliar spays were done starting at 8 April (3rd leaves unfolded, BBCH-13) until 7 June (Berries beginning to touch, BBCH-77). ^2^ Abir, fungicide containing quinoxyfen. ^3^ Heliosulfur, fungicide containing mineral sulfur. ^4^ Means within columns followed by different letters are significantly different (*p* < 0.05, according to Fisher’s LSD K-ratio *t*-test).

**Table 2:** Efficacy of nutrients and fungicides in controlling powdery mildew on the clusters of *Vitis vinifera* cv. Chardonnay at Eshtaol vineyard (trial 2) and at Meron vineyard (trial 3), 2019.

Treatment ^1^	Eshtaol (Trial 2) ^4^		Meron (Trial 3)	
	Incidence (Clusters)	Severity (Clusters)	Incidence (Clusters)	Severity (Clusters)
Control	35 a ^5^	10.3 a	100 a	33.6 a
Top KP+ (1%)	12.7 ab	0.5 b	88 ab	7.4 b
Top KP+ (1%) + Nanovatz (0.1%)	2 b	0.1 b	68.5 bc	4.8 cd
Top KP+ (1%) + TruPhos (0.25%)	4 b	0.2 b	60 c	3.8 d
Abir 250SC ^2^ (0.02%)	0 b	0 b	70.5 bc	5.2 c
Heliosulfur 700SC ^3^ (0.5%)	2.7 b	0.1 b	n.t ^6^	n.t

^1^ In Eshtaol vineyard, for each treatment, five foliar spays were done starting at 27 March (3rd leaves unfolded, BBCH-13) until 5 June (Majority of berries touching, BBCH-79). In Meron vineyard, for each treatment, five foliar spays were done starting at 23 April (5th leaves unfolded, BBCH-15) until 10 June (Berries beginning to touch, BBCH-77). ^2^ Abir, fungicide containing quinoxyfen. ^3^ Heliosulfur, fungicide containing mineral sulfur. ^4^ Disease rating was taken at 19 June 2019 in both Eshtaol and Meron vineyards. ^5^ Means within columns followed by different letters are significantly different (*p* < 0.05, according to Fisher’s LSD K-ratio *t*-test). ^6^ n.t = not tested.

**Table 3:** Efficacy of nutrients and fungicide in mixtures or alternation in controlling powdery mildew on leaves and clusters of *Vitis vinifera* cv. Riesling at Eshtaol vineyard (trial 4), 2020.

Treatment ^1^	Incidence (Leaves) ^4^	Severity (Leaves)	Incidence (Clusters) ^5^	Severity (Clusters)
Control	73.5 a ^6^	26.2 a	98.3 a	55.8 a
Top KP+ (0.5%) + TruPhos (0.25%)	22 b	1.7 b	3.8 b	0.7 b
Top KP+ (1%) + TruPhos (0.25%)	11 bc	0.5 b	0 c	0 b
Top KP+ (1%) + TruPhos (0.25%) + Folicur (0.02%) ^2^	1.3 c	0.2 b	0.6 bc	0 b
Top KP+ (1%) + TruPhos (0.25%)/Folicur (0.02%) ^3^	4 c	0.1 b	0.6 bc	0 b
Top KP+ (1%) + Nanovatz (0.1%)	4 c	0.7 b	1.9 bc	0.3 b
Top KP+ (1%) + Nanovatz (0.1%) + Folicur (0.02%) ^2^	2.7 c	0.1 b	0 c	0 b
Folicur 250EC (0.02%)	1.3 c	0.1 b	1.3 bc	0 b

^1^ For each treatment, five foliar spays were done starting at 14 April (3rd leaves unfolded, BBCH-13) until 3 June (Majority of berries touching, BBCH-79). ^2^ Tank mixture of the macro plus micro nutrients and the fungicide Folicur. ^3^ Alternating applications of the macro plus micronutrients and the fungicide Folicur, starting with first application of the nutrients. ^4^ Disease rating on leaves was taken at 25 May 2020. ^5^ Disease rating on clusters was taken at 25 June 2020. ^6^ Means within columns followed by different letters are significantly different (*p* < 0.05, according to Fisher’s LSD K-ratio *t*-test).

**Table 4:** Efficacy of nutrients and fungicide in mixtures or alternation in controlling powdery mildew on leaves and clusters of *Vitis vinifera* cv. Carignan at Mazkeret-Batya vineyard (trial 5), 2020.

Treatment ^1^	Incidence (Leaves) ^4^	Severity (Leaves)	Incidence (Clusters) ^5^	Severity (Clusters)
Control	98.8 a ^6^	55.8 a	69.4 a	36.3 a
Top KP+ (0.5%) + TruPhos (0.25%)	68.1 ab	10.9 b	29.4 b	10.5 b
Top KP+ (1%) + TruPhos (0.25%)	74.4 ab	12.3 b	26.3 b	4.4 cd
Top KP+ (1%) + TruPhos (0.25%) + Folicur (0.02%) ^2^	26.9 cd	1.8 b	10.0 b	0.5 e
Top KP+ (1%) + TruPhos (0.25%)/Folicur (0.02%) ^3^	63.8 b	16.1 b	25.0 b	9.1 bc
Top KP+ (1%) + Nanovatz (0.1%)	54.4 bc	6.3 b	16.9 b	3.8 de
Top KP+ (1%) + Nanovatz (0.1%) + Folicur (0.02%) ^2^	11.9 d	0.2 b	3.8 b	0.4 e
Folicur 250EC (0.02%)	61.3 b	5.5 b	25.6 b	5.5 cd

^1^ For each treatment, six foliar spays were done starting at 6 April (3rd leaves unfolded, BBCH-13) until 8 June (Majority of berries touching, BBCH-79). ^2^ Tank mixture of the macro plus micro nutrients and the fungicide Folicur. ^3^ Alternating applications of the macro plus micronutrients and the fungicide Folicur, starting with first application of the nutrients. ^4^ Disease rating on leaves was taken at 17 May 2020. ^5^ Disease rating on clusters was taken at 17 June 2020. ^6^ Means within columns followed by different letters are significantly different (*p* < 0.05, according to Fisher’s LSD K-ratio *t*-test).

**Table 5:** List of significantly up-produced metabolites in grapes treated with fertilizers or fungicide and their characteristics. Colored rows differentiates between analyses and cultivars Riesling and Carignan.

**Metabolites Up-Produced in Sprayed Berries (Fertilizers or Fungicide) vs. Untreated Berries**					
**Compound**	**Class/Function**	**Anti-Microbial Effect**	**Antioxidant**	**Wine Quality**	**References**
**cv. Riesling**					
Leu-Arg	Dipeptides	antifungal, plant defense mechanism, disrupting microbial membranes			[36,37,38]
dihydroxy-phenylalanine			antioxidant stability of wines		[39]
Boc-Ser-OH Boc-Gln-OH				impart tastes, including umami, and sweetness	[40,41]
L-gamma-Glutamyl-L-leucine					
Pheophorbide A	Product of chlorophyll breakdown in plants	antiviral, anti-parasite activities	high antioxidant activity		[42,43]
6-tuliposide B	Tuliposides	antifungal, antimicrobial, antibacterial			[44,45,46]
Nocardicin E	Monocyclic β-lactam antibiotics	antibacterial			[47]
**cv. Carignan**					
trans-3-Indoleacrylic acid	Organic acids, Plant growth hormone	antibacterial			[48,49]
Indole	Aromatic organic compound	antifungal (powdery mildew), boosts defense signaling and herbivore resistance			[50,51]
Phytosphingosine 6-hydroxysphing-4E-enine	Sphingoid base	antifungal, Induces systemic acquired resistance, Induces programmed cell death associated with plant defense			[52,53,54]
Limocitrin	Flavonols		antioxidant activity		[55,56]
(±)-(2E)-Abscisic acid	Sesquiterpenes, Phytohormone	regulator in plant biotic defense responses and abiotic stress tolerance			[57,58]
**Metabolites up-produced in fertilizers treated berries vs. fungicide treated berries**					
**Compound**	**Class/function**	**Anti-microbial effect**	**Antioxidant**	**Wine quality**	**References**
**cv. Riesling**					
L-Homoarginine	Non-protein amino acids	antifungal, alkaline phosphatase inhibitor			[59,60,61,62]
2-Deoxy-2-(methacryloylamino)-D-glucopyranose	Carbohydrates	starting materials of bioactive compounds			[63]
6-O-Acetyl-D-glucose	Esters			improve the mouthfeel of wine	[64]
L-2-Hydroxyglutaric acid	Organic acids			conserve wine color, buffer the wine’s pH, impact on bouquet and sourness	[65,66]
(2S)-2-(beta-D-Glucopyranosyloxy)succinic acid					
4-O-Acetyl-D-galacturonic acid					

**Table 6:** Field trials conducted to assess the efficacy of nutrients and fungicides against *E. necator*.

Trial	Year	Location, Region	Treatments ^1^	Grape Cultivar	Age of Vines (Years)	# of Sprays	Date of Bloom ^2^	First Spray	Last Spray
1	2018	Merom-Golan,	Top KP+ (1%) + Nanovatz (0.1%), Top KP+ (1%) + TruPhos (0.25%), Abir (0.02%), Heliosulfur (0.5%), Control untreated	Cabernet Sauvignon	9	6	6 May	8 April (BBCH-13 ^3^)	7 June (BBCH-77)
		Golan							
2	2019	Eshtaol, Judean-Foothills	Top KP+ (1%), Top KP+ (1%) + Nanovatz (0.1%), Top KP+ (1%) + TruPhos (0.25%), Abir (0.02%), Heliosulfur (0.5%), Control untreated	Chardonnay	19	5	25 April	27 March (BBCH-13)	5 June (BBCH-79)
3	2019	Meron, Upper-Galilee	Top KP+ (1%), Top KP+ (1%) + Nanovatz (0.1%), Top KP+ (1%) + TruPhos (0.25%), Abir (0.02%), Control untreated	Chardonnay	19	4	12 May	23 April (BBCH-15)	10 June (BBCH-77)
4	2020	Eshtaol, Judean-Foothills	Top KP+ (0.5%) + TruPhos (0.25%), Top KP+ (1%) + TruPhos (0.25%), Folicur (0.02%), Top KP+ (1%) + TruPhos (0.25%) + Folicur (0.02%) (TM ^4^), Top KP+ (1%) + Nanovatz (0.1%), Top KP+ (1%) + Nanovatz (0.1%) + Folicur (0.02%) (TM ^3^), Top KP+ (1%) + TruPhos (0.25%) + Folicur (0.02%) (Alt ^5^), Control untreated	Riesling	20	5	30 April	14 April (BBCH-13)	3 June (BBCH-79)
5	2020	Mazkeret-Batya, Judean-Foothills	Top KP+ (0.5%) + TruPhos (0.25%), Top KP+ (1%) + TruPhos (0.25%), Folicur (0.02%), Top KP+ (1%) + TruPhos (0.25%) + Folicur (0.02%) (TM ^4^), Top KP+ (1%) + Nanovatz (0.1%), Top KP+ (1%) + Nanovatz (0.1%) + Folicur (0.02%) (TM ^3^), Top KP+ (1%) + TruPhos (0.25%) + Folicur (0.02%) (Alt ^5^), Control untreated	Carignan	20	6	28 April	6 April (BBCH-13)	8 June (BBCH-79)

^1^ Fertilizers were applied with the nonionic surfactant Triton X100 at 0.025%. ^2^ Date of bloom: BBCH-61 = Beginning of flowering: 10% of flowerhoods fallen. ^3^ BBCH-13 = 3rd leaves unfolded, BBCH-15 = 5th leaves unfolded, BBCH-77 = Berries beginning to touch, BBCH-79 = Majority of berries touching. ^4^ TM = Tank Mixture. ^5^ Alt = Alternating applications of Top KP+ (1%) plus TruPhos (0.25%), Folicur (0.02%), Top KP+ (1%) plus TruPhos (0.25%), etc.

## Data Availability

The data presented in this study are available on request from the corresponding author.

## Supplementary Materials

The following supporting information can be downloaded at: https://www.mdpi.com/article/10.3390/plants11070978/s1, Table S1: Determination of must quality and weight of berries of cvs. Riesling and Carignan wine-grapes treated with nutrient mixtures and tebuconazole in 2020 trials. Table S2: Identification of metabolites detected by LC-MS/MS in grape skins of cv. Riesling through MS^1^ and MS^2^. Table S3: Identification of metabolites detected by LC-MS/MS in grape skins of cv. Carignan through MS^1^ and MS^2^.
