# Supplementary material for: Mixtures of Macro and Micronutrients Control Grape Powdery Mildew and Alter Berry Metabolites
Source: Plants (Basel). 2022 Apr 4;11(7):978. doi: 10.3390/plants11070978 (PMC9002579; doi:10.3390/plants11070978)
Supplement: Supplementary file 1 [file plants-11-00978-s001.zip › plants-1632271-supplementary.pdf]

Mixtures of macro and micronutrients control grape powdery mildew and alter berry metabolites

Lior Gur, Yigal Cohen, Omer Frenkel, Ron Schweitzer, Meir Shlisel and Moshe Reuveni

Supplementary material

Table S1. Determination of must quality and weight of berries of wine grapes cvs. Riesling and Carignan treated with nutrient mixtures and Folicur 250EC in 2020 trials.

Treatment	Brix [a]	pH	Berry weight (g) [b]
cv. Riesling (Eshtaol vineyard) [c]			
Control	19.63 ± 0.38a[d]	3.17 ± 0.03a	n.t [e]
Folicur 250EC	19.79 ± 0.82a	3.18 ± 0.02a	n.t
Top KP+ 1% + TruPhos 0.25%	18.44 ± 0.68a	3.21 ± 0.02a	n.t
Top KP+ 1% + Nanovatz 0.1%	18.79 ± 0.90a	3.21 ± 0.01a	n.t
Commercial plot[f]	21.00	3.17	n.t
cv. Carignan (Mazkeret-Batya vineyard) [g]			
Control	21.56 ± 0.32a	3.64 ± 0.03a	1.85 ± 0.050a
Folicur 250EC	20.73 ± 0.19a	3.60 ± 0.01a	1.95 ± 0.052a
Top KP+ 1% + TruPhos 0.25%	20.55 ± 0.68a	3.63 ± 0.02a	2.00 ± 0.057a
Top KP+ 1% + Nanovatz 0.1%	20.62 ± 0.83a	3.63 ± 0.00a	1.99 ± 0.052a
Commercial plot	21.00 ± 0.20	3.74 ± 0.03	n.t

[a] Five berries were collected from the upper and middle part of each of 20 clusters (10 from each side) in each replicate plot.
[b] The weight of each of 50 berries per replicate was determined.
[c] Berries of white grape cv. Riesling in Eshtaol vineyard were collected on August 9, which was 17 days before commercial harvest in the vineyard.
[d] Numbers are means ± standard error of samples for each replicate plot of each treatment. Means within columns followed by different letters are significantly different ($p<0.05$, according to Fisher's LSD K-ratio *t*-test).
[e] n.t = not tested.
[f] Commercial plot= the rest of the vines in the plot adjacent to the trial, which was treated commercially by the grower.
[g] Berries of red grape cv. Carignan in Mazkeret-Batya vineyard were collected on August 30, which was 3 days before commercial harvest in the vineyard.

Table S2. Identification of metabolites detected by LC-MS/MS in skins of wine grape cv. Riesling through MS^1 and MS^2.

Compounds	Molecular Formula	Molecular Weight	RT[a] [min]	MS[1b] (Chemspider)	MS[2c] (FISH)	MS[2] (MzCloud)	Fragments identification (%)
THP(A)	C6 H10 N2 O3	158.06926	1.2	*	*		31.58
Pheophorbide A	C35 H36 N4 O5	592.26860	25.036	*	*		12.96
Nocardicin E	C19 H17 N3 O7	399.10643	1.240	*	*		3.03
N~2~-(4-Aminobenzoyl)arginine	C13 H19 N5 O3	293.14773	1.366	*	*		39.66
L-2-Hydroxyglutaric acid	C5 H8 O5	148.03733	1.3	*	*		40
L-Homoarginine	C7 H16 N4 O2	188.12742	1.177	*	*		66.67
L-gamma-Glutamyl-L-leucine	C11 H20 N2 O5	260.13755	1.333	*	*		68.85
D-(+)-Maltose	C12 H22 O11	364.09839	1.222	*		*	84.4
9-(2-Deoxyhexopyranosyl)-9H-purin-6-amine	C11 H15 N5 O4	281.11115	1.217	*	*		14.02
6-tuliposide B	C11 H18 O9	294.09524	1.274	*	*		30.43
6-O-Acetyl-D-glucose	C8 H14 O7	222.07404	1.206	*	*		72.22
4-O-Acetyl-D-galacturonic acid	C8 H12 O8	236.05341	1.289	*	*		75
2-Deoxy-2-(methacryloylamino)-D-glucopyranose	C10 H17 N O6	247.10565	1.198	*	*		56.76
(2S)-2-(beta-D-Glucopyranosyloxy)succinic acid	C10 H16 O10	296.07461	1.288	*			91.5
cis-Resveratrol	C14 H12 O3	228.07888	10.343	*		*	61.1
ent-Catechin 3-O-gallate	C22 H18 O10	442.09041	9.405	*			25.53

[a] RT= Retention time.
[b] MS^1= Identification carried out through the Chemspider database.
[c] MS^2= Identification carried out through the M/Z cloud database or FISH score algorithm.

Table S3. Identification of metabolites detected by LC-MS/MS in skins of wine grape cv. Carignan through MS^1 and MS^2.

**Compounds**	**Molecular Formula**	**Molecular Weight**	**RT[a] [min]**	**MS^{1b} (Chemspider)**	**MS^{2c} (FISH)**	**MS^2 (MzCloud)**	**Fragments identification (%)**
trans-3-Indoleacrylic acid	C11 H9 N O2	187.06318	6.575	*		*	94.2
Phytosphingosine	C18 H39 N O3	317.29276	17.626	*	*		79.17
6-hydroxysphing-4E-enine	C18 H37 N O3	315.27717	16.877	*	*		90.48
Limocitrin	C17 H14 O8	346.06924	10.088	*	*		31.03
Indole	C8 H7 N	117.0583	6.573	*			15.38
(±)-(2E)-Abscisic acid	C15 H20 O4	264.13596	7.062	*	*		50
	C20 H30 N2 O10	458.18977	8.62	*			
	C21 H34 O9	430.22072	9.757	*			
	C16 H35 N O11	417.22076	6.241	*			
	C21 H34 O9	430.22066	8.192	*			
	C40 H74 N9 O5 P	791.554	29.328	*			
	C18 H39 N O12	461.247	6.856	*			
	C49 H78 N6 O13	958.56182	28.258	*			
	C32 H33 N O18	719.16997	10.093	*			
	C46 H74 N2 O5 P2	796.50911	29.326	*			
	C32 H33 Cl O15	692.15095	10.102	*			
	C32 H26 N4 O11	642.15889	9.692	*			
	C26 H67 N10 P3	612.47484	29.326	*			
	C36 H32 O7 P2	638.16174	10.088	*			

[a] RT= Retention time.
[b] MS^1= Identification carried out through the Chemspider database.
[c] MS^2= Identification carried out through the M/Z cloud database or FISH score algorithm.
